# It's not always easy to buy the idea: strategies, perceptions, and implications of learner-centered teaching in coach education

**DOI:** 10.3389/fspor.2025.1534372

**Published:** 2025-01-28

**Authors:** William das Neves Salles, Lincoln Cruz Martins, Juarez Vieira do Nascimento, Michel Milistetd

**Affiliations:** Sports Pedagogy Laboratory, Postgraduate Program in Physical Education, Federal University of Santa Catarina, Florianópolis, Brazil

**Keywords:** learner-centeredness, constructivism, learning, coach education, sport coaching

## Abstract

**Introduction:**

The study aims to investigate the development of a learner-centered teaching (LCT), a constructivist-based proposal focused on the learner and the learning process, in a university-based coach education program (CEP) through an action research (AR) approach.

**Methods:**

Participants were six students, the researcher and the professor of a Sport Pedagogy course of a public Brazilian university. The classes were taught in a collaborative way between the professor and the researcher, and rubrics were used to guide the LCT development process.

**Results and discussion:**

The proposal was developed gradually and progressively in the course, through diversified teaching strategies that prioritized student interaction and reflection. Facilitators were constantly concerned to ensure that students' perspectives and knowledge were valued during the construction of new teaching content, contributing to align the course with the LCT perspective. While at the beginning of the course the dimensions function of content and role of the teacher were emphasized, in subsequent classes greater attention was devoted to increasing students' responsibility for learning, balance the power between facilitators and students, and promote integrated, authentic assessment processes. To advance the investigated CEP, we recommend the LCT development in other courses of the program, enabling the consolidation of a curricular culture aligned to the constructivist perspective.

## Introduction

Current society, characterized by increasing volatility, uncertainty, complexity, and ambiguity, present educational institutions and programs with the need to move from linear modes of thought to problem solving with synthetic and simultaneous thinking (1). This scenario naturally imposes new demands on sports coaches' education programs (CEPs), where the biggest challenge (and objective) becomes not to cover the content, but to develop the learners’ autonomy in identifying and selecting the information most relevant to their own professional development (2).

Sports coaching, in this sense, has gone through a process of constant legitimation and improvement at a global level, which has implied an increase in the number of CEPs, as well as the re-signification of some standards and/or frameworks (e.g., International Council for Coaching Excellence – ICCE). In fact, CEPs have sought to overcome the deterministic perception of coaching (right vs. wrong) in favor of a more relativistic perspective, in which coaches themselves are positioned at the center of the learning process (3–5).

Thus, a learner-centered perspective has been advocated to improve the quality of CEPs in different contexts (6, 50). The claim of learner-centered teaching is based on constructivist principles, aligning the learner with the learning principles, as explained by McCombs and Whisler (7): “The perspective that couples a focus on individual learners (their heredity, experiences, perspectives, backgrounds, talents, interests, capacities, and needs) with a focus on learning (the best available knowledge on learning and how it occurs and on teaching practices most effective in promoting the highest levels of motivation, learning, and achievement for all learners)” (p. 9).

Studies have demonstrated signals of a shifting paradigm, from an instructor-centered to a more learner-centered approach (6), showing a constant use of active learning methods such as problem-based learning (51), flipped classrooms (8), ethnodrama (3), and the continuous use of reflective practice (9, 10). Even though studies have been demonstrating delivery changes in CEPs in National Governing Organizations (NGOs), there is growing evidence in the university setting (11, 12).

Despite the social recognition of university-based CEPs since they provide the formal initial education of coaches in many countries, universities are still mostly organized under a disciplinary, fragmented, top-down structure (13), which can hinder the implementation of constructivist proposals. There has been a lack of understanding about the operationalization of a constructivist teaching approach and understanding of the role of the former as a facilitator (14, 15, 47). Additionally, students frequently enroll in CEPs with no prior coaching experience, which can result in *a priori*tization of performing well in exams and obtaining a degree rather than learning how to become a coach (12).

A constructivist proposal specially designed for the university context, named Learner-Centered Teaching (LCT), was idealized by Weimer (16) and expanded/systematized by Blumberg (17). This proposal presents an objective and detailed set of dimensions and indicators that guide its operationalization, which can facilitate its implementation and evaluation at the university context. The LCT is organized in five dimensions: function of the content; role of the teacher/instructor; responsibility for learning; balance of power; purposes and processes of assessment. These dimensions are operationalized through a set of components each, resulting in a structure with 29 components that define the LCT. Blumberg (17) has also developed a set of rubrics to diagnose the status of the course and make changes to make it more learner-centered. The rubrics adopt four continuous levels to assess the LCT's degree of implementation: instructor-centered; lower level of transition; higher level of transition; learner-centered (17).

The LCT model cannot be confused with a set of diversified, constructivist teaching strategies. Instead, it is a perspective that seeks to balance the distribution of responsibilities between instructors and students, promoting the development of autonomy and greater participation of students in planning, implementation, and evaluation of the course (17). The instructor, when playing the role of mediator, facilitator, and guide of learning, must present and develop the teaching contents from the previous knowledge, needs and interests of the students, making them more articulated with their academic and professional realities (16). Finally, the LCT's proper implementation requires alignments with the institutional philosophy, the structural conditions available and the students' levels of preparation (17).

While we recognize the challenges posed by the top-down, fragmented, content-based structure of the universities, we believe that a small-scale, bottom-up approach could be an interesting starting point to inspire deeper and more lasting changes in the structure of university-based CEPs and their respective teaching/learning approaches and practices. To our best knowledge, there is a gap in the literature regarding the systematic implementation of the 29 components listed by Blumberg (17) in university-based CEPs through action research (AR). The AR proposal adopted in this study, in turn, is justified mainly to bridge the gap between theory and practice (18) and develop the experiential learning of student coaches (8).

We therefore expect this investigation to contribute to broadening the understanding about the possibilities of implementing the LCT in a university context. Accordingly, the aim of this study is three-fold: (a) To plan and develop an LCT-oriented course in a university-based CEP; (b) to investigate the students' and main researcher's perceptions about the proposal developed and; (c) to identify the potential and limitations of this perspective in the university context.

## Method

### Contextualization

This study was part of a doctoral thesis of which the general objective was to analyze the LCT development process in a Brazilian university-based CEP and was carried out in a Sport Pedagogy course offered to the bachelor's degree in PE of a public Brazilian university. The university has been the subject of recent studies on its CEP curricular organization, with emphasis on the still problematic articulation between its theoretical guidelines and the everyday teaching practices. Recent studies have shown an excessive adoption of prescriptive teaching strategies and the approach of content in a fragmented, decontextualized way when compared to the professional reality of coaching, as well as the lack of supervised reflective practices (19, 20), which reveals inconsistencies between the constructivist conceptual orientations mentioned in the guiding documents (such as the undergraduate program regulation and the Bachelor regulation in Physical Education) and the actual teaching practices in the Bachelor program.

The SP course, specifically, was mandatory and developed in the first semester of the bachelor program through weekly meetings (3 class hours each), totaling 18 meetings and 54 c/h per semester. The course's learning goal was to prepare students to plan and conduct training sessions in team sports. To achieve this goal, classes were organized in three teaching units: (1) Fundamentals of Sports and Sports Pedagogy; (2) Teaching Approaches of Sport; and (3) Coaching Behaviors.

### Participants

At the time of study, the researcher (first author) was a second-year PhD student at the university and assistant teacher in the PE course. He had previous experience as a volleyball athlete and coach (recreational level). His interest in sports coaches’ education and professional development was accentuated by having contact with the LCT literature suggested by the professor.

The professor (second author), PhD in PE, is an associate professor at the university. He had eleven years of teaching experience in higher education (six years teaching SP) and was also involved in teaching and research activities on coach education, coach development and SP. He is a specialist in LCT, coach developer and course designer both in higher education and sports federations.

The researcher and professor had known each other for six years before the study. Their relationship was created and consolidated because both participated in the same research laboratory and shared interest for university-based CEPs. They worked together at the same SP course to conduct a preliminary transformation process towards greater alignment with the LCT principles (21). The professor acted as the researcher's critical friend in the study, participating from the study's conception to the data collection and analysis (22). The classes were taught in a collaborative way between the professor and the researcher. In the classes taught by the professor, the researcher assisted him whenever necessary, so that the class would align with the intended LCT components.

In the first class, the facilitators contextualized the importance and objectives of the course within the CEP, and then explained the intention of developing it throughout the semester from the LCT perspective. After explaining the collaborative nature of this proposal, the facilitators led an introductory activity, in which everyone (facilitators and students) presented themselves to the group of 31 students enrolled. At this time, the facilitators distributed the students into six groups according to some similarities (e.g., sporting experience, academic background) so that they could share their trajectories with each other in more depth. During the activity, the main researcher spoke with each of the groups and invited one student from each to formally participate in the research on a voluntary basis. The invitation was open to everyone in the group so that anyone interested could express their interest. Thus, six students (Box 1) were selected to be the class spokespersons regarding the development of the LCT throughout the semester.

BOX 1 **Students’ biographies and expectations.**

**Table:** 

Student^a^	Gender	Age	Sports experience	Expectations with the course
Tayná	Female	21	Started at 5 years old. Judo and Jiu-jitsu (performance). Keep competing. Working as a sports club coordinator.	To understand the role of the sports coach.
Ahlan	Male	18	Started at 12 years old. Volleyball (performance). Arm injury (break), but keep competing.	To figure out new ways to teach sports.
Felipe	Male	20	Started at 13 years old. Soccer (recreational).	To learn how sport can be taught in different contexts.
Alexandre	Male	18	Started at 5 years old. Soccer (performance). Abandonment because of studies.	To develop knowledge to work with sport at different levels.
Jaqueline	Female	18	Started at 9 years old. Dance (performance). Abandonment because of studies, Previously worked as a dance teacher.	To learn sport teaching methods.
José	Male	21	Started at 12 years old. Surf, Soccer and Tennis (recreational). Previously worked as a Surf coach.	To learn strategies to making sport enjoyable for athletes.

Source: Study Data (2019).

Obs.: ^a^Pseudonyms.

The selection of six students (and not all students in the class) is justiﬁed by the facilitators’ intention to understand in more details the perceptions of certain students regarding the LCT development. Given the course's collaborative nature, the other students, although not formally being the spokepersons, also contributed with suggestions for the general organization throughout the semester, either directly during regular classes or through the class spokespersons.

### Ethical considerations

The study was approved by the university's Ethics Board (n. 2.345802/2017). Before data collection, students were informed about the nature and objectives of the study. The volunteer student involvement was emphasized, including the possibility of withdrawal at any time, without any consequence. Students were notiﬁed that their participation in the research would not affect their grades in the course. After clarification, they agreed to participate and signed an informed consent form.

### Study design and data collection

**Figure 1:**
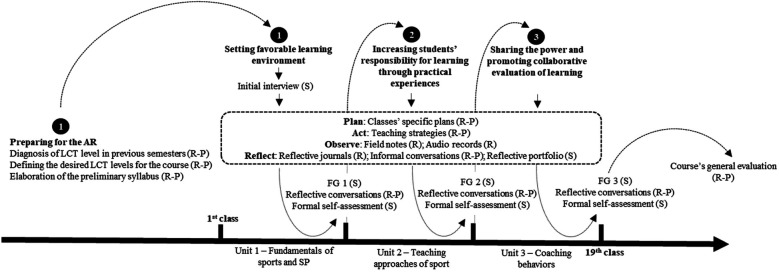
Overall AR process conducted in the study. Source: Study Data (2019). Subtitles: AR, Action research; LCT, learner-centered teaching; (S), Student activity; (R), Researcher-led activity; (P), Professor-led activity; FG, Focus group session.

AR was chosen as methodological approach because it is closely related to the systematic inquiry of the teaching and learning processes and outcomes, with explicit transformational intentions (23). Moreover, AR fits well with the culture of evidence- and theory-based practice at the university setting (24). Therefore, the AR conducted in this study was structured (Figure 1) in a spiral process of planning, acting, observing, and reflecting (25).

Planning started one month before the first class. Both the researcher and professor met to diagnose the LCT level of the course taught in the previous semester. They filled out in detail the five application activities proposed by Blumberg (17) to promote transformations in each dimension. By answering specific questions to each component (e.g., for function of the content—varied uses of content: “Why do students at the level of your course learn the content? Do you explicitly and consistently discuss why students need to learn this content?), the instructor can focus on each individual LCT component, so that they reflect on how that component is being developed in their pedagogical practice, and on the changes that they can implement to make it more aligned with the LCT.

Next, researcher and professor elaborated the course's plan for transformation (17) for each dimension. This activity seeks to make the LCT planning process clearer, since the facilitators can deepen their reflection on the reasons that lead them to develop the LCT in the course, as well as on the constant adaptations that they can make to overcome the possible obstacles.

In the first class, through a peer presentation activity, the facilitators gathered important information about each student (previous sports experiences, general knowledge about SP and expectations about the course). During the first week, the researcher also conducted semi-structured interviews (average duration of 30 min each) with the six selected students to better understand their biography and expectations with the course. The interviews were recorded and fully transcribed by the researcher, who forwarded them to the students to validate the information. The data collected in the presentations and interviews helped the facilitators to have a more accurate idea of the group's profile, which made it possible to adjust the syllabus in order to better contemplate the students’ needs and interests.

**Figure 2:**
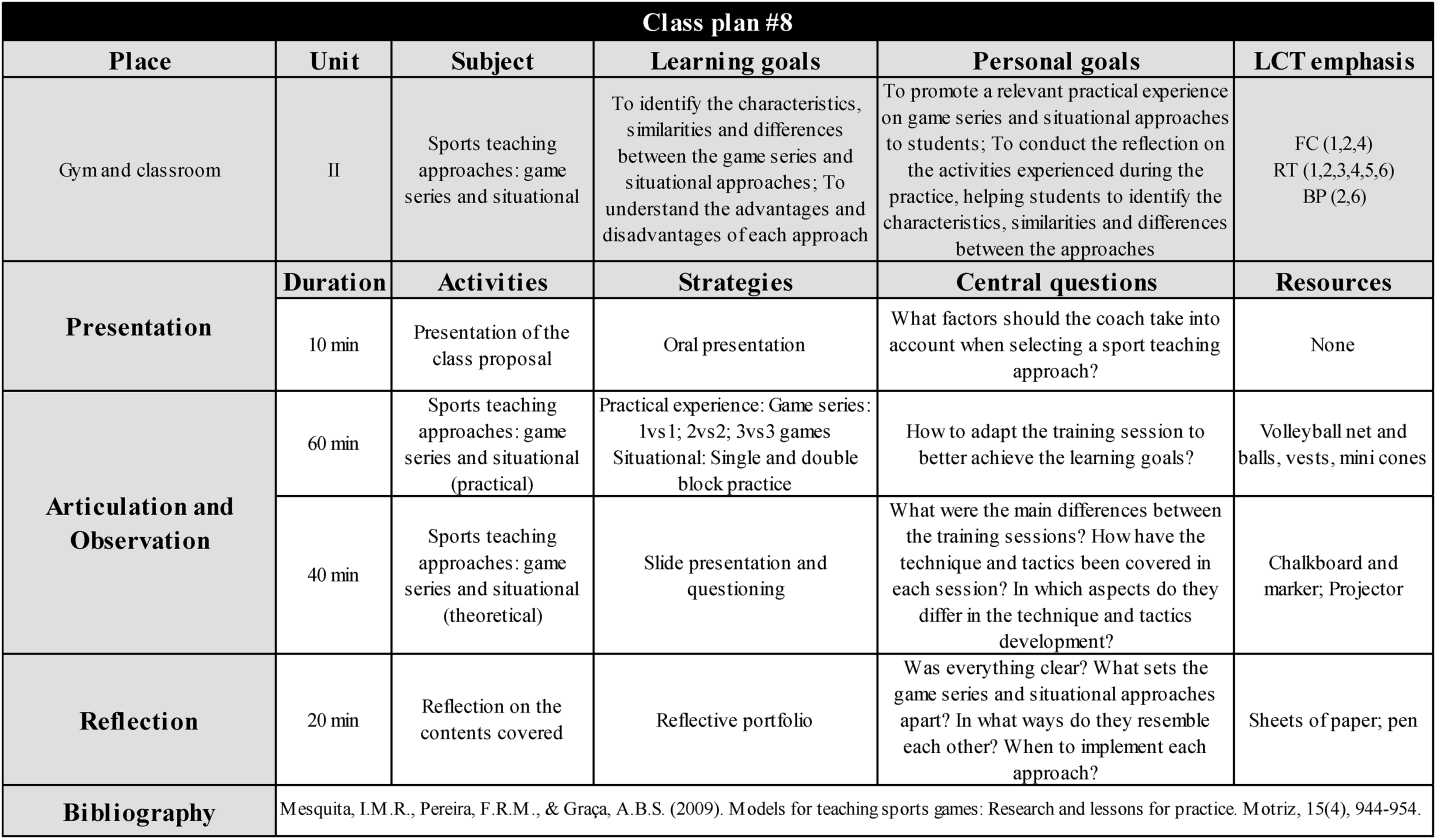
Example of a class teaching plan. Source: Study Data (2019).

Each class had a specific teaching plan (model shown in Figure 2), which organized the class in three moments: (a) Presentation: in this stage, facilitators presented the class proposal (most of the times by posing a question) and the learning objectives to the students, as well as recalled the main contents previously discussed; (b) Articulation and observation: according to the outlined learning goals, facilitators delivered the teaching content by utilizing diverse teaching strategies and, if necessary, made adaptations; (c) Reflection: students reflected on the application of the contents learned in their daily practice, while facilitators reflected on the general effectiveness of the class to achieve the learning objectives. For each class, facilitators aimed to emphasize three to five LCT components, which could be related to one or more dimensions.

Observation occurred during the interventions themselves and was carried out with the help of field notes filled out by the researcher, as well as an audio recorder. Through the notes, researcher sought to identify the students’ reactions to the strategies proposed by the facilitators, as well as insights on aspects that could be discussed with the professor at the end of the class. These observations allowed the facilitators to promote specific changes to increase student involvement. Moreover, the observations brought out some elements for the reflexive notes, carried out by the researcher after each intervention.

Reflection was conducted through a reflective journal filled after each class, which was organized around six generating themes: (a) Class's learning objectives; (b) Researcher's personal goals; (c) Strategies for creating and sustaining the learning environment; (d) Students’ reactions/behavior towards the proposals; (e) Positive and negative aspects of the class; (f) Aspects to maintain and/or improve for the next classes. To ensure greater depth in the reflections, researcher listened to parts of the recordings when answering the questions, to revisit some specific situations in greater detail.

Three focus group sessions with an average duration of 90 min each were conducted by the researcher with the six selected students, which were audio recorded and took place at the end of each teaching unit, after the regular class hours. The purpose of the focus group sessions was to analyze the LCT development through the students' perceptions and discuss the possible next steps for the course. The information collected was literally transcribed and forwarded to students for content validation.

Reflective conversations between the facilitators occurred in between each class, in which they shared their overall perceptions about the last class (teaching strategies adopted, students’ involvement, difficulties faced etc.) and planned the strategies to implement in next interventions. Reflections also occurred at the end of each teaching unit, when facilitators met to discuss their impressions about the AR process development and appropriately adjust the planning of the next teaching unit(s).

### Data analysis

The reflective journals were filled by the first author immediately after each class with the support of Microsoft Word (version 2016; Redmond, WA). When filling them, the researcher sought critical incidents—as described in Kosnik (26)—that inspired deep reflections and some adjustments to the development of the LCT. Afterward, the transcript was sent to the professor for consideration and, after the feedback, it was reviewed by the researcher. The focus groups, in turn, were transcribed *verbatim* and sent to the students for validation. Finally, the reflective conversations between the facilitators were transcribed *verbatim* by the main researcher and forwarded to the professor for validation.

The validated data from the reflective journals, focus groups and reflective conversations were inserted and analyzed by the first author with the support of the *QSR NVivo* software (version 12 Plus; Burlington, MA) using the inductive thematic analysis technique (27), which involves the following six steps. *Familiarization with the data* (1) started during the collection process itself, as the AR approach required the active participation of the researcher in all stages of the process (planning, conducting, observing, and evaluating). At this stage, the researcher took notes about the potential units of meaning derived from the information set. In the *initial coding* (2), the content of the transcripts was segmented into units of meaning. The *identification of emerging themes* (3) was carried out by grouping the units of meaning into higher-order categories based on the presented similarity relations. The stages *reviewing themes* (4) and *naming themes* (5) started from the refinement of the higher-order categories defined in the previous phase. Data from such categories were better specified from the creation of lower-order categories (subthemes), represented by the teaching strategies adopted throughout the course, the student perceptions, and the researcher's reflections on the LCT development. Finally, in the *report production* (6) step, the researcher sought to ensure that the themes and subthemes were supported by text extracts (student speeches; researcher reflections) and aligned with the general narrative about the LCT development in the course.

### Rigor

Researcher's and professor's previous relationship, as well as previous joint work in the same Sport Pedagogy course, allowed both to structure the process in a detailed and collaborative way. Several discussions between them during study planning, data collection, data analysis, and report writing allowed the researcher to expand his analysis through critical reflection (28). Due to the collaborative nature of AR, we also considered students’ perceptions and multiple data sources for LCT development and assessment, such as class plans, observation notes, and reflective journals.

## Results

### Phase 1. Preparing for the AR and setting a favorable learning environment

#### Diagnosing and planning the course's LCT levels

From the initial experience of restructuring the SP course towards the LCT approach (21), we sought to further deepen the focus on the specific components of each LCT dimension, as well as to better systematize the AR cycles. Therefore, the facilitators conducted an application activity (17) to diagnose the course's LCT status at that moment. Next, they elaborated a plan for transformation (17) for planning the LCT level to be attained in each component.

To support the LCT development and evaluation in the course, facilitators applied the rubrics created by Blumberg (17) for each dimension. The rubrics have four continuous levels to assess the degree of LCT development: (1) use of instructor-centered approaches, (2) lower level of transition, (3) higher level of transition, (4) use of learner-centered approaches. The elaboration of the rubrics underwent empirical and specialized validation processes with faculty from different higher education institutions (29).

**Figure 3:**
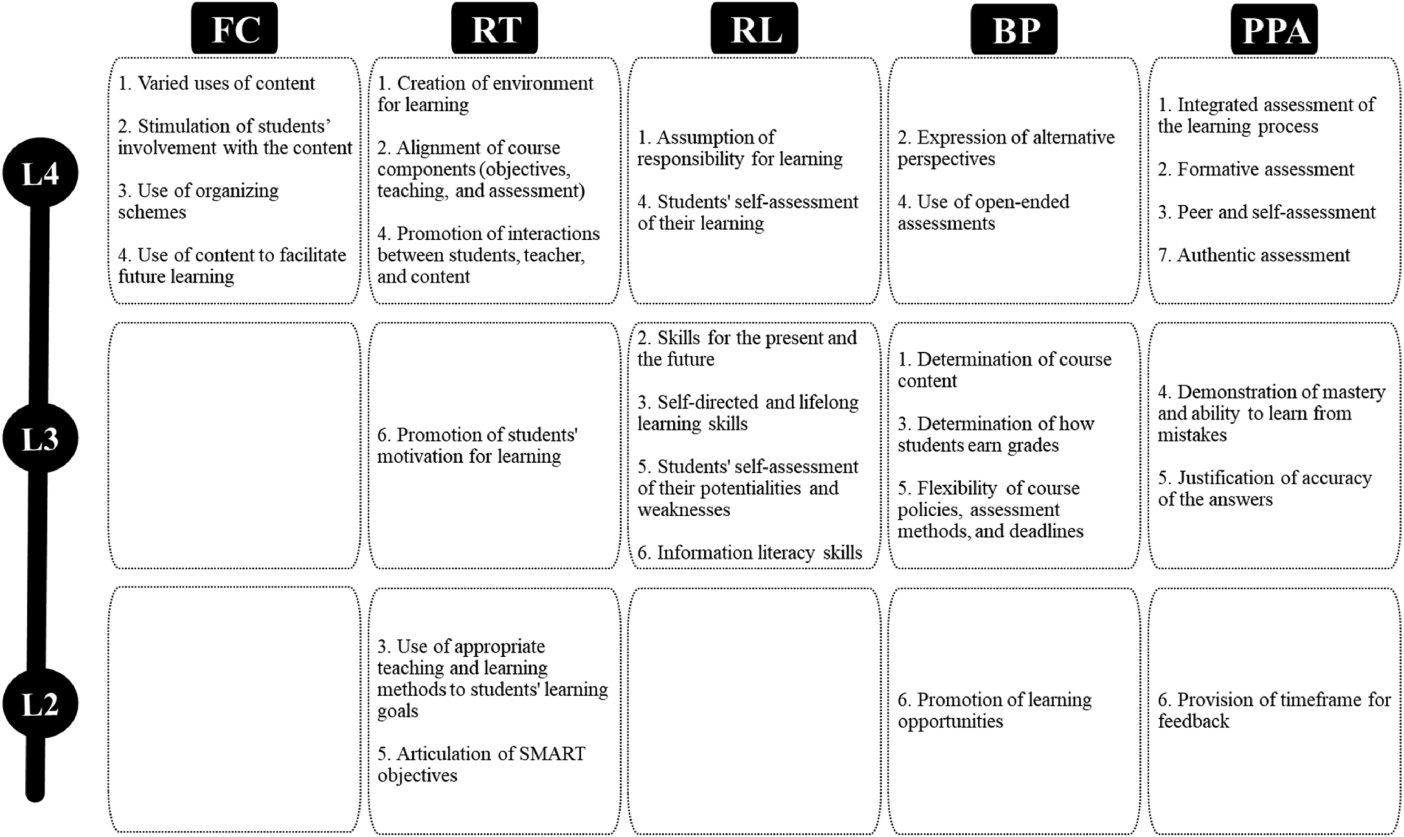
LCT planned levels for the sport pedagogy course. Source: Study Data (2019). Subtitles: Level 2, lower level of transition; 3, higher level of transition; 4, student-centred. FC, Function of the content; RT, Role of the teacher; RL, Responsibility for learning; BP, Balance of power; PPA, Purposes and processes of assessment.

The diagnosis of the LCT level previously developed in the course revealed that most components were still positioned at the lower level of transition, which is why the facilitators chose to develop some of them more effectively during the semester (Figure 3). Considering the introductory nature of the course within the curriculum, as well as the students’ profiles, we chose to emphasize the components of the function of the content and role of the teacher dimensions throughout the semester, while the other components would be emphasized in more specific classes or situations.

To better articulate the AR framework with the teaching contents of the course, we decided to organize the LCT development in three progressive and complementary phases, according to the teaching units. We aimed to involve students from the beginning of the process, so that they realized that their voices would be valued and considered at all phases. At the same time, the facilitators recognized the need to develop the LCT more gradually due to the students’ lack of familiarity with both the LCT and AR proposals, as well as their status at the university context (freshmen).

Specifically, the Sport Pedagogy course's main goal was to create and sustain a positive learning environment. Our intention was to involve students with the content from the very first class by adopting complementary and diversified teaching strategies. In addition, we seek to create connections between the content and the students’ previous experiences as athletes and/or coaches. The planning for the responsibility for learning, balance of power, and purposes and processes of assessment dimensions, in turn, aimed at their development in a more gradual and progressive way, to respect the students’ level of preparation to deal with new responsibilities.

Professor: “The class has 31 students, the majority have now entered the University and they don't know each other. We could explore small group activities, because it will be difficult to give individualized attention to every student. Additionally, this course has only 54 h a semester and occurs once a week, so our time is short.”

Researcher: “I agree with you. As the first teaching unit has a more conceptual orientation, I think we must strive to build the contents based on the students’ realities and experiences, further exploring the dimensions Function of the Content and Role of the Teacher.” (1st reflective conversation)

Considering the course's characteristics and the students’ profiles, the teaching plan established the learning objective of preparing the students to plan and conduct training sessions in team sports. To achieve the objective, the classes were organized in three teaching units: (1) Fundamentals of sport and Sport Pedagogy (15 c/h), whose objective was to identify the guiding conceptions of sports practice, reflecting on the principles of sports initiation and long-term development (classes 1–5); (2) Structural characteristics and didactic-pedagogical approaches for teaching team sports (24 c/h), whose objectives were (a) to distinguish the different didactic-pedagogical approaches employed in sports training and (b) structure the process of teaching-learning sports (classes 6–12); (3) Coaching knowledge and behavior (18 c/h), with the objectives of (a) recognizing the pedagogical role of the coach when teaching sports and (b) identifying the specificities of the different teaching styles adopted by coaches (classes 13–18).

Besides providing interactive teaching strategies (discussions in small and large groups, seminars, role plays, micro-coaching), the teaching plan sought to create opportunities for students to actively participate in the assessment of their own learning. Accordingly, we explored strategies such as filling reflective portfolios after each class, self-correcting written test, and self-assessing coaching performance in practical experiences.

#### Implementing the LCT

**Figure 4:**
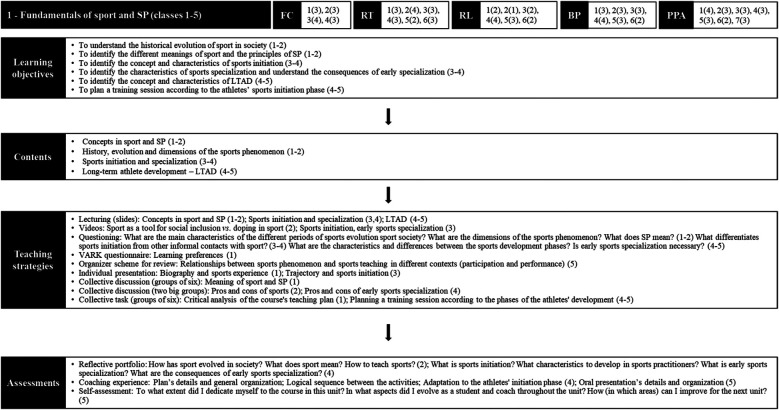
Organizational structure of the sport pedagogy course in unit 1. Source: Study Data (2019). Obs. (1): The researcher was primarily responsible for teaching classes 1, 4, and 5. Obs. (2): LCT dimensions – 1st numbers represent the components themselves; following numbers in parenthesis represent the LCT level reached for each component during the unit. Obs. (3): Learning objectives, contents, teaching strategies, and assessments: numbers in parenthesis represent the classes in which those aspects were developed.

The dimensions function of the content and role of the teacher were emphasized throughout the semester and, especially, during Phase 1, considering that the first teaching unit had a predominantly conceptual focus (Figure 4). Based on diversified teaching strategies such as dramatization, dynamics, group discussions, presentations, and group tasks, students worked on aspects related to the sports origin, evolution and contemporary contextualization, early sports initiation and specialization, and long-term athlete development (LTAD).

#### Students’ perceptions about unit 1

Creating a favorable learning environment. Students valued the facilitators’ concern in creating a positive environment for learning by listening and articulating the students’ perspectives with the content. José acknowledged the fact that “the profs value our little experience”, while Alexandre highlighted the teaching habit of “leaving us comfortable, so that we try to go in the right direction, and you support us”. Ahlan perceived this decision as positive, considering that “everyone comes from a different area, so this approach support a friendly learning environment”.

Articulation of content with previous experiences. Students highlighted that facilitators built the teaching content from the students’ experiences and contributions, which facilitated the recognition and relevance to everyday life and enhanced the students’ interest. Felipe shared that “we can relate what we learn to our experiences”, which was confirmed by Ahlan: “What we have seen here I can relate to other subjects or even with my sporting experience”.

#### Researcher's perceptions about unit 1

*Students’ active engagement* vs. *shyness and lack of participation.* The course's proposal to actively involve everyone in the course's construction was well perceived by most students, as the interest shown through questioning, notes and sharing of experiences and/or personal perspectives was observed. One of the aspects that may have contributed to the students’ high overall engagement may have been the “novelty” itself of entering to the academic environment:

“The use of multiple teaching strategies, with the support of videos and debates, contributed to keep the students’ interest in the class. However, I also identified that the ‘novelty’ factor itself seemed to have contributed to the students paying attention to the teacher during the oral exposition. When we were speaking, the students seemed to perceive us as ‘role models’, as they often nodded in agreement, besides taking notes during our explanation.” (2nd class reflective journal)

While most students were involved in the course's proposal and actively participated in the debates raised by the facilitators, certain classmates seemed to experience difficulties in expressing their perceptions, either in moments of questioning or in group activities. This may have happened because “Probably, only a few had experiences of this nature during high school”, which is why “they still feel shy to better express their perspectives” (2nd class reflective journal).

### Phase 2. Increasing the students’ responsibility for learning through practical experiences

Professor: “We don't have the ideal structure when we teach the first three classes on Mondays, once a week. However, we should keep in mind the fact that when they do not verbalize does not mean that they are not learning. Unit 2 will have a more procedural focus, so they will have more opportunities to experience the application of the concepts in practice.”

Researcher: “Students mentioned in FG that it would be interesting to start covering the next content with practical experiences. Thus, we could start the content of sports teaching approaches at the gym itself, so that we can build the theoretical part based on their perceptions about their practical experiences.” (2nd reflective conversation)

#### Implementing the LCT

**Figure 5:**
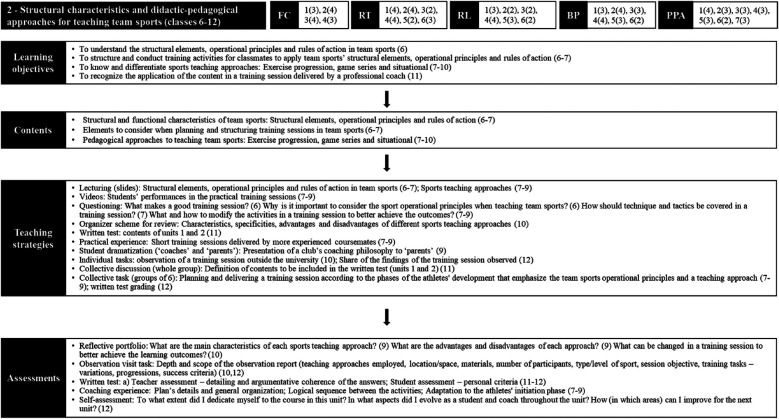
Organizational structure of the sport pedagogy course in unit 2. Source: Study Data (2019). Obs. (1): The researcher was primarily responsible for teaching classes 7, 8 and 10. Obs. (2): LCT dimensions – 1st numbers represent the components themselves; following numbers in parenthesis represent the LCT level reached for each component during the unit. Obs. (3): Learning objectives, contents, teaching strategies, and assessments: numbers in parenthesis represent the classes in which those aspects were developed.

As unit 1 had a more conceptual focus, the objective of unit 2 was to deepen the development of the procedural dimension of sports coaching (Figure 5). Specifically, the structural elements and operational principles of team sports were addressed, as well as some approaches of teaching such as exercise progressions, game series, and situational.

#### Students’ perceptions about unit 2

Content articulation with the coaching practice. Students highlighted the relevance of the contents for the expansion of their own awareness about the coaching intervention. Jaqueline realized that her “sense became much more critical in relation to the training session”, while Ahlan valued the fact of being able to “better understand the different methods to work with different types of athletes and ages”. Moreover, the practical experiences stimulated students to recall their past experiences as sports practitioners, allowing to analyze the positive and negative aspects of such moments in the light of the teaching contents. According to Tayná, “I remembered the trainings that I had in the past, and now I can see how they fit into the approaches we learned in the course. This is helping me a lot to be a good coach, as I can be more critical about what my previous coaches have taught me”.

Difficulties of working in groups. Students indicated that they sometimes did not like working in groups because some ended up dedicating themselves more than others. Some students did not get involved properly and were distracted by other activities unrelated to the class, such as using the cellphone. Tayná and Alexandre expressed their dissatisfaction with this type of strategy because “People have their own times, each has their own problems, so we have no idea what each person's priority is”. Felipe realized that “Each one has a different level of commitment”, and Jaqueline mentioned that, at times, it was difficult to dialogue with classmates in the group due to lack of friendship: “There is the affinity issue within the group, which sometimes distances us a little and makes communication difficult”.

#### Researcher's perceptions about unit 2

Difficulties in time management. As the unit 2 has prioritized the articulation between theoretical and practical aspects of the sports coach's routine, the facilitators had difficulties on certain occasions to properly manage the time of each activity:

“I realized that the organization of large groups during the dynamics, combined with the short time spent for collective reflection, made it difficult for more in-depth debates to take place among its members, and the consequent establishment of a consensus before the information was socialized with the class”. (9th class reflective journal)

### Phase 3. Sharing the power and promoting collaborative assessment of learning

Researcher: “Students are already getting to understand the relationship between the conceptual contents and the sports teaching approaches. However, they are still struggling in managing the training session demands and sharing the responsibilities with their colleagues. I think they are still very attached to the training plan and forget that, perhaps, more important is the training dynamics itself.”

Professor: “This is happening because they are still getting used to planning and conducting training sessions by applying the teaching approaches. They are still getting to know each other, so this lack of intimacy may be making them more insecure to act.” The focus of unit 3 should be more procedural and attitudinal. They need to have more practical experiences as coaches, where they can evaluate their own performance and the collaboration of colleagues in the process”. (3rd reflective conversation)

#### Implementing the LCT

**Figure 6:**
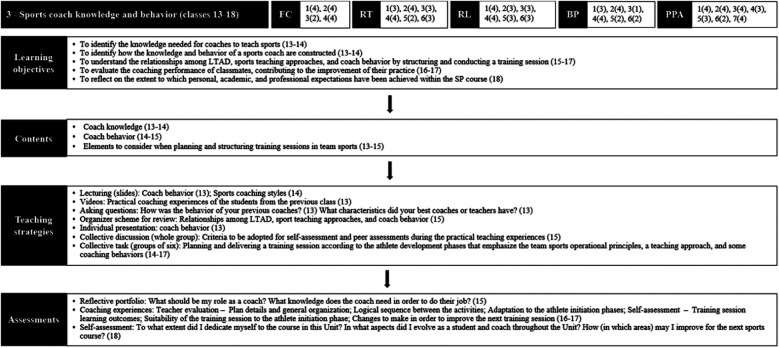
Organizational structure of the sport pedagogy course in unit 3. Source: Study Data (2019). Obs. (1): The researcher was primarily responsible for teaching classes 14, 16, 17, and 18. Obs. (2): LCT dimensions—the 1st numbers represent the components themselves; following numbers in parenthesis represent the LCT level reached for each component during the unit. Obs. (3): Learning objectives, contents, teaching strategies, and assessments: numbers in parenthesis represent the classes in which the specified activities were developed.

Unit 3 brought students closer to the demands faced by sports coaches in their professional practice (Figure 6). Considering that the previous unit focus was to support the class with tools to structure training sessions in a logical and progressive sequence, unit 3 better explored the coaches’ attitudes through more practical coaching experiences with classmates. In the first classes (13th and 14th), facilitators explored the content of coaching behaviors, and in the following four classes, students participated in micro-coaching experiences.

#### Students’ perceptions about unit 3

Developing the ability to adapt to circumstances. The coaching experiences were important to promoting a closer relationship of the students with the reality of sports coaches. Specifically, students valued the ability to adapt to circumstances. According to Felipe, “If we work in groups, as in a technical staff, we will be required to work with people who have had different experiences”. José complemented this by saying that “I figured out that we have to adapt all the time: we have to change because of the level of each one, the number of people we have, and the available materials. There are many factors involved in the coach's practice!”.

Communication and mutual help vs. lack of participation by classmates. Students perceived the group activities in different ways, due to their complexity and unpredictability. For Alexandre, the uncertainty was revealed “because it depends on the people who are in the group, the experience they have with the subject and how interested they are”, which makes the success of the process conditioned to everyone's engagement. José reinforced the importance of developing a sense of interdependence so that everyone assumes their roles in the group: “In group work, we have to think about others, not just your part. You must think that others depend on you; so, if you don't do it, you’re not only prejudicing yourself, but everyone”.

#### Researcher's perceptions about unit 3

Lack of student interest in planning activities. While most of the group showed satisfactory interest and involvement in the unit 3, some students did not seem to be properly involved. During the activity of planning the training sessions for classmates (14th class), researcher identified the lack of respect of some students “when they stay on the cellphone and/or talk to each other about topics other than the class's focus, while they should be helping their classmates in elaborating the training plan”.

Students’ difficulty in evaluating their own performance. Researcher observed the groups’ difficulties in being able to quantify their own performance in class. Even with the collective construction of assessment's criteria and weights during the 14th class, and the provision of a reflective card with indicators that would facilitate the observation of the most relevant aspects to be the assessed, there were problems for establishing consensus:

"The groups’ self-assessment activity revealed that some students are not yet prepared to critically assess themselves, which caused some ‘inflation’ in the grades of certain groups, even after their members have identified limitations in their own performance.” (18th class reflective journal)

## Discussion

This study investigated the development of an LCT-based proposal in a university-based CEP and diagnose the students’ and the main researcher's perceptions about the proposal developed. Student involvement was stimulated in all teaching units, although in different ways.

In the early classes, facilitators diversified teaching strategies and conducted debates on controversial topics or dilemmas of the sports coach's daily practice to maintain the group engagement. In units 2 and 3, the interest was sustained by the greater approximation with the coach's reality, either through the practical experiences (in which students acted as participants), or assuming the coach's role with classmates.

It is noteworthy that the use of different teaching strategies is fundamental in the teaching-learning process (30), contributing to the increase of motivation and engagement of learners, as well as providing opportunities for the development of critical thinking and meaningful learning, enhancing the transfer and application of knowledge in different contexts and situations (31–33).

In addition, as for the facilitators, one of the objectives was to create and maintain favorable learning environments in unit 1, as well as to build closer relationships with students. This approach was emphasized to gain the trust of students and encourage the constant exchange of information and experiences, supporting the proper development of LCT.

Students positively perceived the facilitators’ efforts to create and maintain a supportive learning environment. They valued the fact that their previous sports experiences had been the object of active discussion in the classroom, as well as the articulation of these experiences with theoretical and practical elements of coaching. By relating the contents to their own trajectories, the students were able to perceive greater meaning in the practice of sports coaching, which allowed them to analyze it in a more critical and broader way.

The promotion of a safer, less intimidating learning environment provides a more active posture and increase the interest of learners, reverberating this posture to the questions and answers in the learning environment (32, 34). In addition, experiential learning is highly valued in constructivist teaching, since it has greater potential to awaken students’ motivation for the content, as well as to encourage the establishment of personal meanings and reflections based on what has been experienced (8, 35). In this process of student experimentation, teacher supervision and support are very important (49), as they can help students reflect more deeply and in detail on their roles in the experience (8, 48).

As for the initial emphasis on the function of the content and role of the teacher dimensions, it is justified because they are easily manipulated and require few substantial changes in the general structure of responsibilities and powers usually present in university courses. In fact, the gradual and progressive approach is recommended (16, 17) to respect the students’ learning rhythms and needs.

In this logic, the activities can be revisited several times throughout the semester, being problematized and practiced in progressively more complex ways at each opportunity (36). There is also an importance in the non-linearity of content during the learning process, corroborating the improvement of critical thinking and decision-making and real-life problems, as well as the transfer of knowledge to the field of action (32, 37).

Regarding the responsibility for learning, it was also developed progressively. Emphasis was given to the component of promoting the self-assessment of students’ learning (level 4 in all units), developed throughout the semester through reflective portfolios and self-assessments. At the beginning of the semester, facilitators decided to reduce students’ responsibility for being newcomers to the higher education environment. Throughout the semester, the gradual approximation of the class with the professional reality of the sports coach (practical experiences on sports teaching approaches; planning, conduction and evaluation of teaching experiences) required greater dedication, engagement and responsibility from the students.

By assuming greater responsibility for their own learning, students can benefit from exercising greater control and power over academic-professional development. In addition to increasing motivation (38), greater responsibility stimulates students to develop complementary and interdisciplinary skills, such as problem solving, peer evaluation, and self-reflection, besides the connection with classmates and professors (17). Finally, when students understand that they are also responsible for the quality of the formative process, they can experience changes in their mentality and start socializing useful content, experiences, and practices with the group (39).

On the other hand, students don't always like/want to take on more responsibilities. In our study, some students had difficulty actively participating in the process of collaborative construction of training plans with their peers. In addition, they sometimes felt intimidated and/or shy in the face of the facilitators’ invitation to express opinions and propose solutions to problems in sports training. In fact, it is more comfortable for students to adopt a passive role for their own learning, because in this way their main attribution is to memorize and reproduce the content taught by the specialist (teacher). In addition, the fact that they are presented with a different pedagogical approach from what they are used to can interfere with the engagement of learners, and the role of facilitators is important in the feasibility of pedagogical change in teaching in the long term (40, 41).

As for the balance of power, it was especially emphasized through the component expression of alternative perspectives (level 4 in all units). From the initial classes, students were encouraged to share their previous experiences with sports practice and their perspectives on the contents covered. The evaluations (reflective portfolios, self-evaluations, observation report, written test, teaching experiences), because they are open, also allowed students to put their perspectives in evidence and use arguments to justify their position. The components that determine how students get grades and flexibility were most encouraged in the final part of the course, which featured more classroom debates about the dilemmas faced by athletic trainers in their routines.

It is important that the sharing of power does not occur automatically and irrationally, but in a dynamic and gradual way, considering the progressively higher levels of maturity and autonomy presented by the students (16, 17). In this sense, the teacher continues to make certain important decisions about the teaching-learning process, but students also have opportunities to participate in decision-making about specific aspects of the program, such as deadlines for achieving learning objectives, types of tasks to be performed to achieve objectives, and forms of assessment (16, 17). It should be noted that, as students are generally deprived of opportunities to assume greater power in higher education, any experience in this regard can make a big difference in students’ motivation and learning process (32, 40, 42).

Regarding the purposes and processes of assessment dimension, the development took place mainly through formative evaluations and integrated into the learning process (level 4 in all units). After each didactic content, the students filled out reflective portfolios, in which they reflected on the content learned and its practical usefulness in everyday life. In the task of preparing a report regarding the visit to the sports club (12th class), the facilitators allowed the students to correct the problematic or insufficient aspects observed and to resend the updated version for a new evaluation, which promoted the demonstration of mastery and ability to learn from mistakes and reinforced the formative character of the evaluation practices. Peer evaluation, self-evaluation, and authentic evaluation were specially developed in the last unit, especially through practical teaching experiences. The authenticity of the assessment was expressed in the student's involvement in the process of planning, conducting, and evaluating the training sessions, while peer evaluation (first teaching experience) and self-evaluation (second teaching experience) took place immediately at the end of the respective training session given.

The formative assessment is commonly used by student-centered educators because its emphasis is on the process of gradually developing student self-awareness about their learning and autonomy (43). With regard to authentic assessment, it is recommended to bring students closer to the contexts of professional practice (17), and its application is very common in academic disciplines that seek to bring students closer to professional practice environments (8, 44), as well as in curricular internships (35). The results of these assessments indicated that students can develop more understanding of the complexity and challenges of professional practice, as well as closer relationships with individuals working in these contexts (8, 35). The importance of a range of assessments is also emphasized, as they help to achieve learning goals, facilitate the appropriation of new knowledge, and promote a deeper understanding of problem solving (32, 45, 46).

Finally, as the course was organized in 54 h/c, distributed in single classes of 3 h/week, it was difficult to sustain the higher level of LCT in certain components. Specifically, the facilitators observed that the components of the function of the content and role of the teacher dimensions were more sustained when compared to the others, which reached this level only at specific moments. The determination of the component of obtaining students’ grades, for example, was discussed only in two moments: during the presentation of the teaching plan (1st class) and in the definition of the evaluation criteria and respective weights for the teaching experiences (14th class).

Another limitation refers to the fact that the study was conducted in a single Sport Pedagogy course. Due to its own pedagogical character, this course favored the application of a constructivist teaching proposal. Moreover, we acknowledge that the research was facilitated by the previous relationship built between the researcher and the professor. The shared ministration of an academic course may not correspond to the reality faced by many university professors, who need to accomplish this task without any direct peer collaboration.

### Practical recommendations for the teachers/instructors

(1) Before classes begin, put yourself in your students’ shoes as you develop your course's transformation plan. Perform the activities that will be proposed to them beforehand, to get an idea of the motivations and difficulties they can trigger. If possible, ask other students at the university how they would feel doing them, or even simulate doing certain activities with them.
(2) In the first classes, present the LCT framework in detail, and its implications for the general structuring of the course. Justify the importance of this experiment for the students’ professional development and make it clear that it will be a laborious process for everyone. At the same time, encourage them to notice and comment on the differences between the LCT proposal adopted in your course and proposals adopted in other courses of the CEP.
(3) Initially, emphasize the function of the content and the role of the teacher dimensions. The operationalization of its components depends more on the professor's initiative, so this will be more natural and comfortable for everyone.
(4) Get to know your students. Explore their past experiences with sport, motivations and learning expectations. Organize activities that, at some point, match their profiles.
(5) Create a positive learning environment based on interaction and reflection. Propose collective activities in which students discuss different perspectives and build solutions to problems. Also explore individual and collective reflective activities, in which students identify their potential and areas for improvement. Make students feel comfortable and encouraged to ask. Avoid judging their opinions as right or wrong; instead, help them see the positive and negative consequences of their opinions.
(6) Be flexible in your teaching approach. Use different teaching strategies, as each student learns better in a certain way. Adapt the course structure whenever necessary, seeking to align the course objectives with the students’ learning needs. Take a centralizing role, depending on the needs of the situation. Remember that the journey towards LCT is not linear.
(7) Develop multiple forms and perspectives of assessment. Involve students in building assessment criteria and encourage them to assess their peers and themselves in certain activities.
(8) In the face of conflicts or difficulties (e.g. lack of collaboration between students, shyness), reinforce the justification for adopting the LCT. Listen to student feedback and change something if necessary. But do not abandon the original idea of the LCT proposal and make it clear that students need to face difficulties to evolve and to become more autonomous in their learning journey.
(9) Share your experiences with your supervisor and departmental colleagues whenever possible. Even small initiatives to improve some specific components of the LCT are valid and have the potential to inspire similar initiatives in other courses.
(10) Keep systematic records (e.g., reflective journals) of your LCT implementation process, highlighting its strengths and weaknesses at the personal and institutional levels, to facilitate the organization of thought and obtain valuable evidence that can later be shared with other stakeholders.

### Practical recommendations for the university-based CEPs

(1) To build a philosophy and establish an internal policy aligned with the LCT principles, which includes the elaboration of both curricular documents and institutional evaluation criteria appropriate to the teaching-learning practices carried out at the CEP. Make it clear that this is not an “all or nothing” approach, but rather an incremental one that must be appropriately contextualized to the needs of each course.
(2) To promote peer-to-peer teacher education activities based on the LCT approach. The CEP educators themselves, as they adopt the LCT approach, may help their less experienced colleagues in this process.
(3) To provide regular times and spaces (e.g., seminars, workshops) for the faculty to exchange experiences regarding good LCT practices.
(4) To produce comprehensible teaching material (with examples of good practices) based on the LCT principles, in order to highlight the importance and facilitate the adoption of this approach by less familiar teachers.
(5) To present the LCT principles and involve students in the systematic evaluation of the LCT developed in the CEP courses.

### Final considerations

The LCT implementation showed to be a complex process, which was dynamically built from several debates and reflections between the researcher, the instructor, and the students. Although the intention was to develop the process gradually and progressively, it did not take place in a linear manner. At certain times (e.g., shyness and lack of participation of some students when planning activities and self-assessing their learning), there was a need for greater intervention and guidance from facilitators to convince students of the importance of taking greater responsibility for their own professional development.

Prior to implementing the LCT, we were already aware of the time constraints to develop the proposal, as well as the potential difficulties arising from the students’ profiles (freshmen). To facilitate the transition towards higher levels of LCT, we sought to develop the process gradually, progressively (with special emphasis on the dimensions FC and RT), and systematically, to ensure that all 29 components would be properly covered. However, despite the facilitators’ intention to reach the highest possible LCT level, it was not possible to devote so much attention to certain components due to limitations such as the course's workload and the weekly class distribution.

The list of 29 components provides an important guide to support LCT practice toward the highest levels of student autonomy. Nonetheless, we sometimes struggled to define the criteria to be considered when evaluating the level obtained at a given component (e.g., Is one open-ended assessment enough to consider that we reach the LC level on BP? How many times do students need to self-evaluate their learning throughout a teaching unit for it to be considered a LC level on RL?). These aspects suggest the cautious and contextualized interpretation of the findings.

Finally, to advance the LCT development in university-based CEPs, we highlight the importance of combining a top-down approach led by institutional administrators—so that the LCT culture embed the whole program, with a bottom-up approach led by professors in their courses—which enables the differentiation of strategies and processes according to the courses’ specificities.

## Data Availability

The original contributions presented in the study are included in the article/Supplementary Material, further inquiries can be directed to the corresponding author.
